# Editorial: Process Data in Educational and Psychological Measurement

**DOI:** 10.3389/fpsyg.2021.793399

**Published:** 2021-12-03

**Authors:** Hong Jiao, Qiwei He, Bernard P. Veldkamp

**Affiliations:** ^1^Department of Human Development and Quantitative Methodology, University of Maryland, College Park, MD, United States; ^2^Educational Testing Service, Princeton, NJ, United States; ^3^Department of Learning, Data Analytics and Technology, Faculty of Behavioral Management and Social Sciences, University of Twente, Enschede, Netherlands

**Keywords:** process data, psychological assessment, data mining, educational assessment, computer based assessment

The increasing use of computer-based testing and learning environments is leading to a significant reform on the traditional form of measurement, with tremendous extra available data collected during the process of learning and assessment (Bennett et al., 2007, 2010). It means that we can learn and describe the respondents' performances not only by their responses, but also their responding processes, in addition to the response accuracy in the traditional tests (Ercikan and Pellegrino, 2017).

The recent advances in computer technology enhance the convenient collection of process data in computer-based assessment. One such example is time-stamped action data in an innovative item which allow for the interaction between a respondent and the item. When a respondent attempts an interactive item, his/her actions are recorded, in the form of an ordered sequence of multi-type, time-stamped events. These sorts of data stored in log files, referred to as *process data* in this book, provide information beyond response data that typically show response accuracy only. This additional information holds promise to help us understand the strategies that underlie test performance and identify key actions that lead to success or failure of answering an item (e.g., Han et al., 2019; Liao et al.; Stadler et al., 2019; He et al., 2021; Ulitzsch et al., 2021a; Xiao et al., 2021).

With the availability of process data in addition to response data, the measurement field is becoming increasingly interested in borrowing additional auxiliary information from the responding process to serve different assessment purposes. For instance, recently researchers proposed different models for response time and the joint modeling of responses and response time (e.g., Bolsinova and Molenaar; Costa et al.; Wang et al.). In addition, other process data such as the path collected based on eye-tracking devices (e.g., Zhu and Feng, 2015; Maddox et al., 2018; Man and Harring, 2021), action sequences in problem-solving tasks (e.g., Chen et al.; Tang et al., 2020; He et al., 2021; Ulitzsch et al., 2021b), and processes in collaborative problem solving (e.g., Graesser et al., 2018; Andrews-Todd and Kerr, 2019; De Boeck and Scalise, 2019), are also worthy of exploration and integration with product data for assessment purposes.

This Research Topic (formed in this edited e-book) intends to explore the forefront of responding to the needs in modeling new data sources and incorporating process data in the statistical modeling of multiple possible assessment data. This edited book presents the cutting-edge research related to utilizing process data in addition to product data such as item responses in educational and psychological measurement for enhancing accuracy in ability parameter estimation (e.g., Bolsinova and Molenaar; De Boeck and Jeon; Engelhardt and Goldhammer; Klotzke and Fox; Liu C. et al.; Park et al.; Schweizer et al.; Wang et al.; Zhang and Wang), cognitive diagnosis facilitation (e.g., Guo and Zheng; Guo et al.; Jiang and Ma; Zhan, Liao et al.; Zhan, Jiao et al.), and aberrant responding behavior detection (e.g., Liu H. et al.; Toton and Maynes).

Throughout the book, the methods for analyzing process data in technology-enhanced innovative items in large-scale assessment for high-stakes decisions are addressed (e.g., Lee et al.; Stadler et al.). Further, the methods for the extraction of useful information in process data in assessments such as serious games and simulations were also discussed (e.g., Liao et al.; Kroehne et al.; Ren et al.; Yuan et al.). The interdisciplinary studies that borrow data-driven methods from computer science, machine learning, artificial intelligence, and natural language processing are also highlighted in this Research Topic (e.g., Ariel-Attali et al.; Chen et al.; Hao and Mislevy; Qiao and Jiao; Smink et al.), which provide new perspectives in data exploration in educational and psychological measurement. Most importantly, the models presenting the integration of the process data and the product data in this book are of critical significance to link the traditional test data with the new features extracted from the new data sources. Meanwhile, the papers included in the book provide an excellent source for data and coding sharing, which entails significant contributions to the applications of the innovative statistical modeling of assessment data in the measurement field.

The book chapters demonstrate the use of process data and the integration of process and product data (item responses) in educational and psychological measurement. The chapters address issues in adaptive testing, problem-solving strategy, validity of test score interpretation, item pre-knowledge detection, cognitive diagnosis, complex dependence in joint modeling of responses and response time, and multidimensional modeling of these data types. The originality of this book lies in the statistical modeling of innovative assessment data such as log data, response time data, collaborative problem-solving tasks, dyad data, change process data, testlet data, and multidimensional data. Further, new statistical models are presented for analyzing process data in addition to response data such as transition profile analysis, the event history analysis approach, hidden Markov modeling, conditional scaling, multilevel modeling, text mining, Bayesian covariance structure modeling, mixture modeling, and multidimensional modeling. The integration of multiple data sources and the use of process data provides the measurement field with new perspectives to solve assessment issues and challenges such as problem-solving strategy, cheating detection, and cognitive diagnosis.

An overview of all the papers included in this Research Topic is summarized in Table 1 with respect to their key features. The scope of the Research Topic can be classified into five major categories:

1. leveraging process data to explore test-takers' behaviors and problem-solving strategies,
2. proposing joint modeling for response accuracy and response times,
3. proposing new statistical models on analyzing response processes (e.g., time-stamped sequential events),
4. advancing cognitive diagnostic models with new data sources, and
5. using data streams in estimating collaborative problem-solving skills.

**Table 1:** An overview of papers collected in this Research Topic.

**References**	**Areas of advancement**	**Data types**	**Statistical approaches**	**Assessment domains**
**Leveraging process data to explore test-takers' behaviors and strategies**				
Ren et al.	Exploring multiple goals in interactive problem-solving items	Extracted response process variables, correctness of responses	Cluster analysis, logistics, and least-squares regression	Interactive problem-solving in PISA 2012
Engelhardt and Goldhammer	Proposing a validity research that uses processing times to provide both convergent and discriminant validity evidence for the construct interpretation of reasoning and reading ability scores	Response data, response times	MLR estimator (maximum likelihood estimation with robust standard error)	PIAAC 2012 literacy assessments
Stadler et al.	Exploring successful and unsuccessful strategies with process data in complex problem-solving items	Response process data, correctness of responses	N-grams model	Interactive problem-solving items
Lee et al.	Exploring response times in complex simulation-based tasks to understand test-takers' interactions	Response data, response times	Cluster analysis and hierarchical framework for joint modeling item responses and response times	Interactive problem-solving items
Toton and Maynes	Detecting examinees with pre-knowledge in experimental data with conditional scaling of response times	Item scores, response times	Cluster analysis, factor analysis	Simulation study and empirical study in GRE quantitative testing
Arieli-Attali et al.	Understanding test-takers' choices using hidden Markov modeling of process data	Response data, answer change, item difficulty	Hidden Markov model	Self-adapted tests
Qiao and Jiao	Using data mining techniques in analyzing process data and making comparisons among machine-learning algorithms in exploring problem-solving items	Extracted response process variables, correctness of responses	Multiple machine learning algorithms: supervised techniques (CART, gradient boosting, random forest, and SVM), unsupervised techniques (SOM, k-means)	Interactive problem-solving in PISA 2012
Liu H. et al.	Exploring test-takers' problem-solving strategies with a modified multilevel mixture IRT model	Extracted response process variables, correctness of responses	Modified multilevel mixture IRT model, latent class analysis	Interactive problem-solving in PISA 2012
Liao et al.	Exploring sequential patterns in problem-solving items and relationship with individual differences in background variables	Extracted response process variables, response data, background variables	N-grams model, feature selection model, regression analysis	PIAAC 2012 problem-solving in technology-rich environment
**Joint model for response accuracy and response times**				
Zhan, Jiao et al.	Proposing a joint model for multidimensional abilities and multifactor speed	Response data, response times	Joint modeling of response and response time, exploratory factor analysis	Simulation study and empirical study in computer-based math assessment (PISA 2012)
Costa et al.	Proposing a joint model for item response and time-on-task to increase the precision of ability estimates	Response data, response times	Multidimensional latent model for response and response time	Interactive problem-solving in PISA 2012
Guo et al.	Proposing a joint model for a speed-accuracy tradeoff hierarchical model based on cognitive experiment	Response data, response times	Bayesian MCMC algorithm, speed-accuracy hierarchical model	Simulation study and empirical study in Raven's Standard Progressive Matrices
Klotzke and Fox	Proposing a Bayesian modeling framework for response accuracy, response times, and other process data variables	Response data, response times, extracted response process variables	Bayesian covariance structure models	Simulation study and empirical study in PIAAC 2012 cognitive assessments
Kroehne et al.	Proposing a parameterized joint model of response data and response time to detect invariance by gender and mode between computer-based and paper-based tests	Response data, response times	Bivariate generalized linear IRT model framework (B-GLIRT)	PISA 2012 and PISA 2009 reading assessments
De Boeck and Jeon	An overview of models for joint modeling of response times and response accuracy in cognitive tests	Response data, response times	Multiple response models and joint models of response data and response times	Literature review
Wang et al.	Modeling response time and responses in multidimensional health measurement	Response data, response times	Multidimensional-graded response model, hierarchical joint model of responses and response times	Health measurement
Zhang and Wang	Proposing a mixture learning model that utilizes the response times and response accuracy in learning progression	Response data, response times	Diagnostic classification model framework, Bayesian estimation	Simulation study and empirical study in a computer-based learning environment
Bolsinova and Molenaar	Proposing a joint model for response accuracy and response times with consideration on non-linear conditional dependence	Response data, response times	Joint model for quadratic conditional dependence, joint model for multiple-category conditional dependence, indicator-level non-parametric moderation method	Simulation study and empirical study in high-stakes arithmetic assessment
**Statistical model on response process**				
Smink et al.	Therapeutic change process research through multilevel and text mining	Life narratives textual data and response data	Multilevel models, text mining	Epidemiologic Studies Depression Scale and life narratives (CES-D)
Schweizer et al.	Investigating how the major outcome of a confirmatory factor investigation is preserved when scaling the variance of a latent variable by the various scaling methods	Scaling data	Multiple confirmatory factor analysis	Simulation study and empirical study in Multitrait-Multimethod (MTMM) design
Liu C. et al.	Proposing a model with a leakage parameter to better characterize the item leaking process and develop a more generalized detection method by monitoring responses of test-takers	Response data	Generalized linear model for detection, leakage simulation model	Simulation study and empirical study in operational computerized adaptative testing
Park et al.	Proposing a multidimensional IRT approach for dynamically monitoring ability growth in adaptive learning systems	Response data, response times	Multidimensional IRT	Simulation study and web-based learning platform
Chen et al.	Proposing an event history analysis approach to predict duration and outcome of solving a complex problem by making use of process data	Time-stamped sequential events data, correctness of responses	Regression model	Interactive problem-solving in PISA 2012
**Advancement in cognitive diagnostic model with process information**				
Guo and Zheng	Comparing termination rules for variable-length CD-CAT from the information theory perspective	Response data, test construction variables	Multiple cognitive diagnostic models	Simulation study
Jiang and Ma	Proposing a model to integrate differential evolution optimization into the EM framework in the log-linear cognitive diagnostic model estimation	Response data	Log-linear cognitive diagnostic model with EM algorithm, differential evolution	Simulation study and empirical study in assessment of a health profession
Zhan, Liao et al.	Proposing a joint testlet cognitive diagnostic model for paired local item dependence using response time and response accuracy	Response data, response times	Joint testlet cognitive diagnosis modeling	PISA 2015 computer-based math assessment
**Using data streams for estimating collaborative problem-solving skills**				
Hao and Mislevy	Characterizing interactive communications in collaborative problem-solving using a conditional transition profile approach	Conversations collected in a computer-based collaborative problem-solving platform	Conditional transition profile, cluster analysis	Collaborative problem-solving platform
Yuan et al.	Assessing collaborative problem-solving competence by extracting indictors from process stream data and modeling dyad data	Process stream data in collaborative problem solving, response data	Multidimensional Random Coefficients Multinomial Logit Model (MRCMLM)	Collaborative problem-solving platform adapted from a problem-solving task in PISA 2012

The above categorization focused on each paper's core contribution though some papers can be cross-classified. The papers' key findings and advancements impressively represent the current state-of-the-art methods in the field of process data analysis in educational and psychological assessments. As topic editors, we were happy to receive such a great collection of papers with various foci and submit these publications right as digital assessments are booming. The papers collected in this Research Topic are also diverse in data types, statistical approaches, and assessment with an extensive scope in both high-stake and low-stake assessments, covering research fields in education, psychology, health, and other applied disciplines.

As one of the first comprehensive books addressing the modeling and application of process data, this e-book has drawn great attention since its debut was cross-loaded on three journals in *Frontiers in Psychology, Frontiers in Education*, and *Frontiers in Applied Mathematics and Statistics*. With 29 papers from 77 authors, this book enhances interdisciplinary research in fields such as psychometrics, psychology, statistics, computer science, educational technology, and educational data mining, to name a few. As highlighted on the e-book webpage, (https://www.frontiersin.org/research-topics/7035/process-data-in-educational-and-psychological-measurement#impact) on November 13, 2021, this e-book has accumulated 115,069 total reviews and 17,940 article downloads since the Research Topic project launched in 2017. This number keeps growing on a daily basis. The diversified demographics provide convincing evidence that the papers in this book reached the global research community, addressing the critical issues of statistical modeling of multiple types of assessment data in the digital era. This book is just on time to provide tools and methods to shape this new measurement horizon.

As more and more data are being collected in computer-based testing, process data will become a very important source of information to validate and facilitate measuring response accuracy and provide supplementary information in understanding test-takers' behaviors, the reasons of missing data, and links with motivation studies. There is no doubt that there is high demand of such research in the large-scale assessment, both high-stake and low-stake, as well as in the personalized learning and assessment to tailor the best source and methods to help people learn and grow. This book is a timely addition to the current literature on psychological and educational measurement. It is expected to be applied more extensively in educational and psychological measurement, such as in computerized adaptive testing and dynamic learning.

## Author Contributions

All authors listed have made a substantial, direct, and intellectual contribution to the work and approved it for publication.

## Funding

QH was partially supported by the National Science Foundation grants IIS-1633353.

## Conflict of Interest

The authors declare that the research was conducted in the absence of any commercial or financial relationships that could be construed as a potential conflict of interest.

## Publisher's Note

All claims expressed in this article are solely those of the authors and do not necessarily represent those of their affiliated organizations, or those of the publisher, the editors and the reviewers. Any product that may be evaluated in this article, or claim that may be made by its manufacturer, is not guaranteed or endorsed by the publisher.
